# Immediate care for victims of sexual violence: Number 9 – 2025

**DOI:** 10.61622/rbgo/2025FPS9

**Published:** 2025-11-11

**Authors:** Rosires Pereira de Andrade, Fernanda Garanhani de Castro Surita, Aline Veras Morais Brilhante, Robinson Dias de Medeiros, Cristião Fernando Rosas, Helena Borges Martins da Silva Paro, Marla Niag dos Santos Rocha, Olimpio Barbosa de Moraes, Sara de Pinho Cunha Paiva, Stenia dos Santos Lins, Marcia Sacramento Cunha Machado, Rivaldo Mendes de Albuquerque, Jose Paulo de Siqueira Guida, Zelia Maria Campos, Marli Camara Abelha

**Affiliations:** Universidade Federal do Paraná Curitiba PR Brazil Universidade Federal do Paraná, Curitiba, PR, Brazil.; Universidade Estadual de Campinas Campinas SP Brazil Universidade Estadual de Campinas, Campinas, SP, Brazil.; Universidade Federal do Ceará Fortaleza CE Brazil Universidade Federal do Ceará, Fortaleza, CE, Brazil.; Universidade Federal do Rio Grande do Norte Natal RN Brazil Universidade Federal do Rio Grande do Norte, Natal, RN, Brazil.; Hospital e Maternidade Escola de Vila Nova Cachoeirinha São Paulo SP Brazil Hospital e Maternidade Escola de Vila Nova Cachoeirinha, São Paulo, SP, Brazil.; Universidade Federal de Uberlândia Uberlândia MG Brazil Universidade Federal de Uberlândia, Uberlândia, MG, Brazil.; Universidade Federal da Bahia Salvador BA Brazil Universidade Federal da Bahia, Salvador, BA, Brazil.; Universidade de Pernambuco Recife CE Brazil Universidade de Pernambuco, Recife, CE, Brazil.; Universidade Federal de Minas Gerais Belo Horizonte MG Brazil Universidade Federal de Minas Gerais, Belo Horizonte, MG, Brazil.; Universidade Federal do Rio Grande do Norte Natal RN Brazil Universidade Federal do Rio Grande do Norte, Natal, RN, Brazil.; Universidade Federal da Bahia Salvador BA Brazil Universidade Federal da Bahia, Salvador, BA, Brazil.; Universidade de Pernambuco Recife PE Brazil Universidade de Pernambuco, Recife, PE, Brazil.; Universidade Estadual de Campinas Campinas SP Brazil Universidade Estadual de Campinas, Campinas, SP, Brazil.; Universidade Federal do Amazonas Manaus AM Brazil Universidade Federal do Amazonas, Manaus, AM, Brazil. Universidade Estadual do Amazonas, Manaus, AM, Brazil.; Universidade Federal Fluminense Niterói RJ Brazil Universidade Federal Fluminense, Niterói, RJ, Brazil.

## Abstract

Prompt care for victims of sexual violence is essential in the implementation of measures to prevent pregnancy resulting from violence and sexually transmitted infections, and to assess and monitor serological follow-up and gather evidence for possible criminal proceedings.

In the absence of a referral service, any health service should take the appropriate initial measures and refer the victim to the referral service (if there is one) as quickly as possible.

Referral services for immediate care for victims of sexual violence allow victims to be followed up for a longer period of time. However, acute care should be provided by any health unit to avoid care delays.

There is no need for any legal complaint or registration of a police report for providing healthcare to victims of sexual violence. However, victims should be informed about their right to report the assault at any time, so an appropriate legal and police investigation can be carried out. It is important to address this topic during medical care.

All services providing care to victims of sexual violence must be open and receiving victims continuously, without the need for other health units or a police authority.

Obstetrician/gynecologists are often the professionals responsible for caring for victims of sexual violence and must be familiar with the initial care protocols for these patients.

## Recommendations

Care should be provided in a private location to avoid exposing the victim and accompanied by another health professional.

Initial attentive listening and privacy offered to the victim of sexual violence together with adequate records: these are essential for clinical care and monitoring of the case, preventing the patient from having to retell the story, which can lead to revictimization.

Detailed collection of data on sexual violence in medical records, preferably with representations of the human body for an accurate description of the injuries.

Prophylaxis based on the time of aggression and exposure to bodily secretions, prioritizing the maximum provision of prophylactic measures in the event of inaccurate information.

## Background

The World Health Organization (WHO) defines sexual violence as any sexual act, attempted sexual act, unwanted sexual comments or advances, or acts of trafficking, or otherwise directed against a person's sexuality through coercion, which can be perpetrated by anyone, regardless of their relationship to the victim, in any setting, including but not limited to the home and workplace.^(1)^ Sexual violence occurs when consent to sexual intercourse is not obtained, considering that the clear and actively expressed consent is essential and can never be assumed. In addition, certain conditions may determine a temporary inability to consent, such as the use, intentional or unintentional, of psychoactive substances or medical conditions. Furthermore, consent is considered null and void when a person under 14 years of age or with moderate or severe cognitive impairment is involved. Therefore, a wide range of sexually violent acts carried out without appropriate consent in different circumstances and environments, including those listed by the WHO, are grouped under the name of sexual violence, as follows:^(1,2)^

- Marital or partner rape;
- Rape by strangers;
- Systematic rape during armed conflicts;
- Unwanted sexual advances or sexual harassment, including asking for sex in exchange for favors;
- Sexual, mental or physical abuse of people with disabilities;
- Sexual abuse of children;
- Forced marriage or cohabitation, including child marriage;
- Denial of the right to use contraceptive methods or adopt other protective measures against sexually transmitted infections (STIs);
- Forced abortion;
- Violent acts against the sexual integrity of women, including female genital mutilation and mandatory virginity inspections;
- Forced prostitution and human trafficking for sexual exploitation.

Rape is a type of sexual violence, defined by the WHO as non-consensual penetration of the vulva, mouth or anus using the penis or another body part or an object. An attempt to do so is known as "attempted rape". The rape of a person by two or more perpetrators is known as gang rape.^(1,3,4)^

Sexual violence is a global problem present in all cultures, countries and social classes.^(5)^ As gender-based violence,^(4)^ it has deep links with issues of race/ethnicity, class, sexuality, gender identity, age, ability status, citizenship status and nationality.^(6)^ Although it affects mainly women, it is noteworthy that people from other social groups are also at higher risk of sexual violence, such as LGBT people – binary or otherwise.^(7,8)^ Sexual violence is considered a major violation of human rights,^(4,9)^ with short, medium and long-term repercussions for the health and well-being of women and their families, in addition to major social and economic costs.^(4,9–12)^ Therefore, sexual violence constitutes a public health problem^(4)^ that requires an active position from the health system to achieve effective and sustainable responses of assistance to victims and coping with the problem.^(13)^

Brazilian Federal Law Number 12.015/2009^(14)^ removes sexual crimes from the chapter on crimes against customs and includes them in the chapter on crimes against the person and crimes against sexual dignity. The law now provides for a specific classification of the crime of rape against a vulnerable person in its Article 217-A, defined as the act of "having sexual intercourse or practicing another lewd act with a minor under 14 (fourteen) years of age".

The most common crimes against sexual dignity are:

"Rape

Art. 213 – Forcing someone, through violence or serious threat to have sexual intercourse or to practice or allow another lewd act with him/her.

(…)

Sexual assault through fraud

Art. 215 – Having sexual intercourse or engaging in a lewd act with someone through fraud or other means that prevents or hinders the free expression of the victim's will".^(14,15)^

Immediate healthcare for a person who is a victim of sexual violence is a medical emergency hence, it must be provided as quickly as possible. The aim of immediate care is to establish prophylactic measures for pregnancy resulting from rape and prevention of bacterial and viral infections; promote assessment and monitoring of serology for STIs; and allow the production of evidence for possible future criminal proceedings. The International Federation of Gynecology and Obstetrics (FIGO) launched the 2018 FIGO Global Declaration on the Elimination of Violence Against Women,^(16)^ which commits to supporting efforts to address the problem, and was followed by the publication of a protocol by the Brazilian Federation of Gynecology and Obstetrics Associations (Febrasgo), contributing to the immediate care of victims of sexual violence.^(17,18)^

## How to structure an immediate care service for victims of sexual violence?

Although the creation of referral services allows for better monitoring of victims, any health service must promote immediate embracement, referring them to the referral service as quickly as possible or, in the absence of one, instituting initial measures. Health measures do not depend on communication to the police authorities, and the victim must be informed of their rights during clinical care. The care service must be open and receiving victims on their own demand or through referral by other health units or by police authorities themselves.

## What changes should be implemented in the structuring of services?

- Infrastructure: Private environment, preferably in a separate room, whether in the emergency room, Basic Health Unit (UBS) or any other institution where care is provided to avoid overexposure of the victim. The use of jargon or acronyms that may identify the space reserved for the care of victims of sexual violence should be avoided. It is important that the gynecologist be accompanied by at least one other health professional as a way of avoiding intimidation of the victim of sexual violence, who is often in a situation of physical and emotional vulnerability.
- Attentive listening: The gynecologist should try to make the victim feel as safe and comfortable as possible, directing the conversation to establish important information about the violence, such as: a) the time of the violence; b) the place of the violence; c) the forms of violence suffered (sexual, physical, moral, etc.); d) the characterization of sexual violence (anal, vaginal, oral penetration; exposure to secretions; presence of physical injuries); e) the characteristics of the aggressor.
- Recording in medical records: All information obtained from the victim must be recorded so that it can be accessed by other professionals involved in the care, preventing the victim from having to pass on information previously given, which could lead to revictimization. The information should preferably be recorded in electronic medical records to ensure greater data security. However, the absence of electronic medical records should not serve as a barrier to accessing care. Proper recording of the mechanisms of violence, exposure to fluids, findings in physical examination, among other information, is essential for defining the clinical care to be instituted, as well as for legal purposes, since the medical record may be used as evidence in the course of any judicial investigation.
- Physical examination: During the physical examination, it is strongly recommended that the victim of sexual violence be accompanied by a person of her choosing. In addition, the gynecologist must be accompanied by another health professional. The physical examination should begin with a general assessment of the victim's body, paying close attention to bruises, injuries or other dermatological lesions anywhere on the body. The pelvic examination should include an assessment of the genital and anal region, especially when there is a report of vaginal or anal penetration. During the speculum examination, if possible, the secretions found should be collected and a sample sent for laboratory evaluation, which may include bacterioscopy of vaginal secretions, culture or molecular tests for STIs (chlamydia, gonococcus and trichomonas) and fungal culture. The sample should also be stored on filter paper for future legal investigations and DNA identification. Any lacerations identified should be treated surgically using an appropriate technique. If there are clinical signs suggesting genital infections, treatment should be started immediately and the results of laboratory tests should not be awaited. The physical examination should be described in detail in the medical record, including the description and location of the lesions found, the pelvic contents, as well as any genital or anal lesions.
- Mandatory notification form: For epidemiological purposes, in addition to recording information in the medical record, it is necessary to fill out a form in the Notifiable Diseases Information System (SINAN) in triplicate. In this way, the three spheres of government are enabled to conduct the investigation process through a computerized network and have support for analyzing the care provided, allowing decision-making. If the victim is a child or adolescent, the form must also be forwarded to the Child and Adolescent Guardianship Council or the Child and Youth Court, in accordance with the Child and Adolescent Statute.^(19)^
- Laboratory tests: The purpose of collecting blood is to identify the current serological status of the victim of sexual violence and monitor possible serological changes resulting from exposure to violence. Serological collection for HIV, hepatitis B, hepatitis C and syphilis is mandatory when treating victims of sexual violence, not only during the first consultation, but also during follow-up after the treatment is instituted. Early implementation of antimicrobial and antiviral measures can substantially reduce the possibility of infections such as HIV, hepatitis B, hepatitis C, syphilis, chlamydia, trichomonas and gonococcus. For serological monitoring, it is recommended to repeat the serological tests at around 45 and 90 days after exposure. If both results are negative, contamination resulting from sexual violence is ruled out.
- Prophylactic treatment: The decision to institute prophylaxis for pregnancy and bacterial or viral infections involves the time after exposure to sexual violence. In cases where immediate care occurs within 72 hours after the violence (third day after the violence), emergency contraception, antimicrobial prophylaxis and post-exposure prophylaxis for HIV should be provided. In cases where healthcare is sought between the third and fifth day after exposure, emergency contraception is recommended, knowing that its effectiveness is progressively less, depending on the time elapsed after exposure, in addition to prophylaxis for bacterial infections. If the victim seeks healthcare after the fifth day of exposure, only serology collection and scheduling of follow-up appointments should be carried out. All recommended prophylaxis should be instituted at the first appointment, avoiding the victim having to travel to different services, in addition to missing follow-up appointments. The institution of prophylaxis depends only on exposure to violence, regardless of serological results or clinical evidence of infection:
- Emergency contraception: It should be prescribed to all victims of sexual violence who have received care up to the fifth day after exposure with certain or uncertain contact with semen. Victims who use a highly effective contraceptive method, such as an intrauterine device – IUD (copper or hormonal) or etonogestrel implant – may be exempted from taking an emergency contraceptive. In all cases, a pregnancy test should be performed before the patient takes the emergency contraceptive. The first-choice medication is a single dose levonorgestrel 1.5 mg (two 0.75 mg tablets or one 1.5 mg tablet). In cases where the patient vomits after taking the medication, the full dose should be offered again. This regimen has no contraindications other than pregnancy, and can even be prescribed to people with a history of hematologic or thromboembolic disorders. The TCu 380A IUD is an emergency contraceptive method that may also be offered in the context of sexual violence. Its provision should be discussed with the patient if she is sexually active, has no contraindications to this method, and regarding its availability at the specific health service. Intrauterine devices are effective methods of emergency contraception and should be made available to women seeking emergency contraception. Given the risk of an STI after sexual violence, the IUD can be inserted up to five days after the sexual assault for clinically eligible women. The greater risk of STIs after rape should be considered if the copper IUD is used.^(20)^
- HIV prophylaxis: Recommended in cases of sexual violence that occurred up to 72 hours after immediate care, where there was vaginal or anal penetration, regardless of whether there was ejaculation or not. In cases where there was oral penetration with ejaculation, the risks and benefits should be discussed with the victim, especially when there are injuries on the oral mucosa and according to the serological status of the aggressor, if known, and the victim's desire to receive prophylaxis, since the risk of contamination in this exposure is low. Post-exposure prophylaxis for HIV is not recommended in cases where there was certainly oral penetration without ejaculation, as well as condom use during the violence, if the aggressor is known to be HIV positive, or in cases of chronic sexual abuse by the same aggressor. In cases where there is doubt regarding the dynamics of the aggression, the high risk of ejaculation should always be considered, and consequently, prophylaxis as well. Prophylaxis should be instituted within the first 72 hours after the violence and should be maintained for 28 days. The collection of the following tests is recommended: complete blood count, creatinine, urea and liver function. These blood tests should be repeated halfway through the treatment. The preferred regimen consists of: tenofovir 300 mg + lamivudine 300 mg + dolutegravir 50 mg in a single daily dose for 28 days.^(21)^
- Prophylaxis for bacterial infections: Treatment should be instituted as soon as possible after exposure to violence and involves the prevention of infections by syphilis, gonorrhea, chlamydia and trichomonas. The preferred regimen consists of: benzathine penicillin 2.4 million IU intramuscularly – IM (1.2 million IU for each gluteal muscle) + ceftriaxone 500 mg IM + azithromycin 1 g + metronidazole 2 g. All antibiotics should be taken in a single dose. In the case of people allergic to benzathine penicillin, the use of azithromycin 2 g is recommended. In the case of a pregnant patient allergic to benzathine penicillin, desensitization to the drug is recommended, followed by its prescription.^(17,18)^
- Hepatitis B prophylaxis: Prophylaxis should be considered in all cases where exposure to semen is suspected or confirmed and also involves assessing the victim's previous vaccination status. In cases where vaccination status is confirmed, i.e., three doses of previous hepatitis B vaccination, this prophylaxis is not indicated. In other contexts of uncertain vaccination or incomplete vaccination schedule, vaccination and specific immunoglobulin for hepatitis B should be offered.^(17,18)^
- Human papillomavirus (HPV) prophylaxis: Vaccination against HPV for victims of sexual violence was instituted by the Brazilian Ministry of Health and should be offered to people who do not have a complete vaccination schedule. The vaccine should be administered immediately (or as soon as possible) after the violence. A single dose is recommended, in accordance with the most recent national protocols. In cases where the HPV vaccine is not available at the initial care location, the victim of sexual violence should be referred for vaccination at the UBS with a report containing the specific code for the disease (ICD) to avoid revictimization of the patient.^(17,18)^

## The importance of transdisciplinarity

Sexual violence is a medical problem, but also a legal and psychological one; therefore, it is important that the teams involved in care are composed of professionals trained to understand the various dimensions of this phenomenon. When structuring services, it is suggested that gynecologists provide initial care. However, the presence of professionals specialized in mental health in the follow-up, e.g., psychiatrists and psychologists, who will be able to prevent and treat mental events such as post-traumatic stress disorder, is essential. In addition, nursing professionals should be involved in care, creating an environment of embracement and monitoring of victims in the various stages of treatment. It is also essential to include social service professionals, who are equipped to educate victims about their legal rights in the face of sexual violence. The services should interact with other public institutions, such as the local primary care network, so that the transfer of care of victims can occur after a certain period of follow-up (between three and six months), as well as with legal agencies, such as local women's police stations. This allows for a better understanding on the part of both parties, ensuring chains of custody of evidence and facilitating the exchange of information in the context of investigations or legal proceedings. Finally, it is important that organized civil society movements, such as women's movements, non-governmental organizations and organizations that protect individual rights, are familiar with the services, allowing for rapid referral and appropriate guidance for women victims of violence.

## Final considerations

Given the importance of universal care for victims of sexual violence, the services should adapt this protocol to local realities, ensuring access to at least pregnancy and STI prevention measures, which avoids unwanted pregnancies and the issue of legal abortion, which is the subject of a wide range of questions in Brazilian society. Treatment measures should consider any report of sexual violence by the victim, whose story should be valued and not questioned or ridiculed. Detailed data collection in electronic medical records is recommended, which shall be accessed only by other professionals involved in the care. Doubtful cases of exposure to bodily secretions should receive the maximum possible treatment, given the risk of contamination. Multidisciplinary care should be encouraged in the creation of services to care for victims of sexual violence, which should also establish a follow-up proposal for victims that includes serological surveillance for a period of at least 90 days after the assault. Guidance on the patient's legal rights must be provided by the healthcare professionals involved in the care, but it is up to the patient, if of legal age, to decide whether or not to seek criminal prosecution, and her decision must be respected.

## National Commission Specialized in Sexual Violence and Pregnancy Interruption Provided for by Law of the Brazilian Federation of Gynecology and Obstetrics Associations (Febrasgo)

President:

Rosires Pereira de Andrade

Vice-president:

Fernanda Garanhani de Castro Surita

Secretary:

Aline Veras Morais Brilhante

Members:

Robinson Dias de Medeiros
Cristião Fernando Rosas
Helena Borges Martins da Silva Paro
Marla Niag dos Santos Rocha
Olimpio Barbosa de Moraes Filho
Sara de Pinho Cunha Paiva
Stenia dos Santos Lins
Marcia Sacramento Cunha Machado
Rivaldo Mendes de Albuquerque
Jose Paulo de Siqueira Guida
Zelia Maria Campos
Marli Camara Abelha
